# A Modified Hyrax Assembly for Intrusion of a Traumatically Extruded Incisor: An Innovative Technique

**DOI:** 10.7759/cureus.49938

**Published:** 2023-12-04

**Authors:** Pinaki Roy, Poonam Sharma, Harpreet Singh, Pranav Kapoor, Raj Kumar Maurya

**Affiliations:** 1 Orthodontics and Dentofacial Orthopedics, Burdwan Dental College and Hospital, Bardhaman, IND; 2 Orthodontics and Dentofacial Orthopedics, Employees’ State Insurance Corporation (ESIC) Dental College and Hospital, Delhi, IND; 3 Dentistry, Central Government Dental Unit, Army Dental Corps, Dehradun, IND

**Keywords:** trauma, orthodontic, molar tube, incisor intrusion, hyrax expander

## Abstract

This case report demonstrates an innovative technique involving concomitant correction of a traumatic extrusive luxated tooth, the mobility of which was being aggravated by anterior occlusal contacts, along with transverse rapid maxillary expansion to capitalize on the advantage of residual growth and simplify the need for comprehensive fixed orthodontic appliance. By incorporating a molar tube into the acrylic splint of the bonded Hyrax expander adjacent to the buccal surfaces and parallel to the buccal cusps of the maxillary first molars, effective intrusion of traumatically extruded upper incisor was achieved concomitantly using a modified intrusion arch during the passive stabilization period after expansion, thereby reducing treatment time. This enabled the immediate correction of extruded tooth and reduced the overall treatment duration and the complexity of post-expansion fixed mechanotherapy, improving compliance and uplifting the self-esteem of the patient. The modified bonded Hyrax assembly can serve as a versatile interim appliance for the simultaneous management of a variety of orthodontic problems such as crowding, spacing, and incisor proclination without compromising the basic integrity of the bonded assembly.

## Introduction

Extrusive traumatic injuries resulting in the loss of arch length, tooth malposition, and malocclusion present a therapeutic challenge in orthodontics [1]. Successful treatment of such problems poses a greater difficulty over time due to the possibility of ankylosis or tooth loss which can be aggravated under constant antagonist occlusal pressure. In such situations, a combination of simultaneous orthodontic treatment mechanics for the correction of a multitude of associated problems is needed. Correction of simple problems such as anterior crossbite or midline shift during the passive stabilization period after expansion using a modified Haas expander assembly has recently been reported by Caldas et al. [2]. Here, we describe a simple and effective modification of bonded Hyrax assembly for use as an adjunct in the intrusion of a traumatically extruded tooth.

## Case presentation

**Figure 1:**
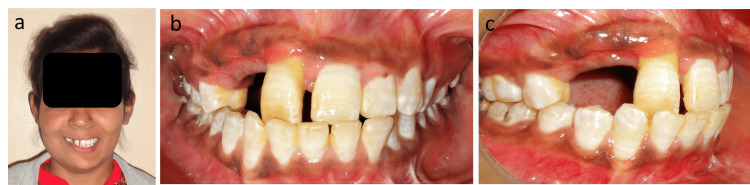
(a) Pretreatment frontal photograph. (b) Pretreatment intraoral frontal view showing a Class III incisal relationship with the traumatic extruded maxillary right central incisor. (c) Pretreatment intraoral right lateral view.

**Figure 2:**
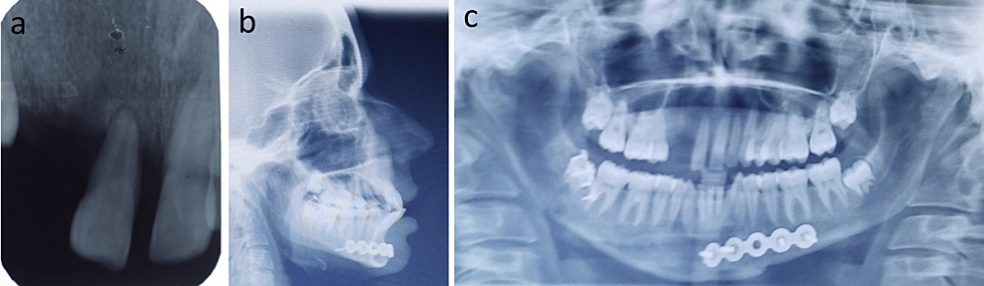
Pretreatment radiographs: (a) intraoral periapical X-ray, (b) lateral cephalogram, and (c) orthopantomagram.

A 14-year-old girl sought treatment for traumatically extruded maxillary right central incisor and avulsed maxillary right lateral incisor, canine, and first premolar (Figures 1A-1C). The patient reported a history of trauma six months back with no corrective measures taken in the immediate post-traumatic period. The extruded incisor displayed elongation along with deviation in axial inclination and abnormal mobility. Clinical examination revealed continuous occlusal shearing force on the extruded tooth from an antagonist, potentially aggravating/hampering the prognosis of survival. There was no evidence of root fracture or pathology as well as no calcification. The vitality of the tooth was confirmed based on thermal and electronic pulp testing. The patient had a Class III incisal relationship, a Class I skeletal relationship with a hyperdivergent growth pattern, and inadequate space to accommodate the maxillary teeth (Figures 2A-2C). The model analysis revealed a constricted maxillary arch by 4 mm with compensated mandibular constriction in the buccal segments.

**Figure 3:**
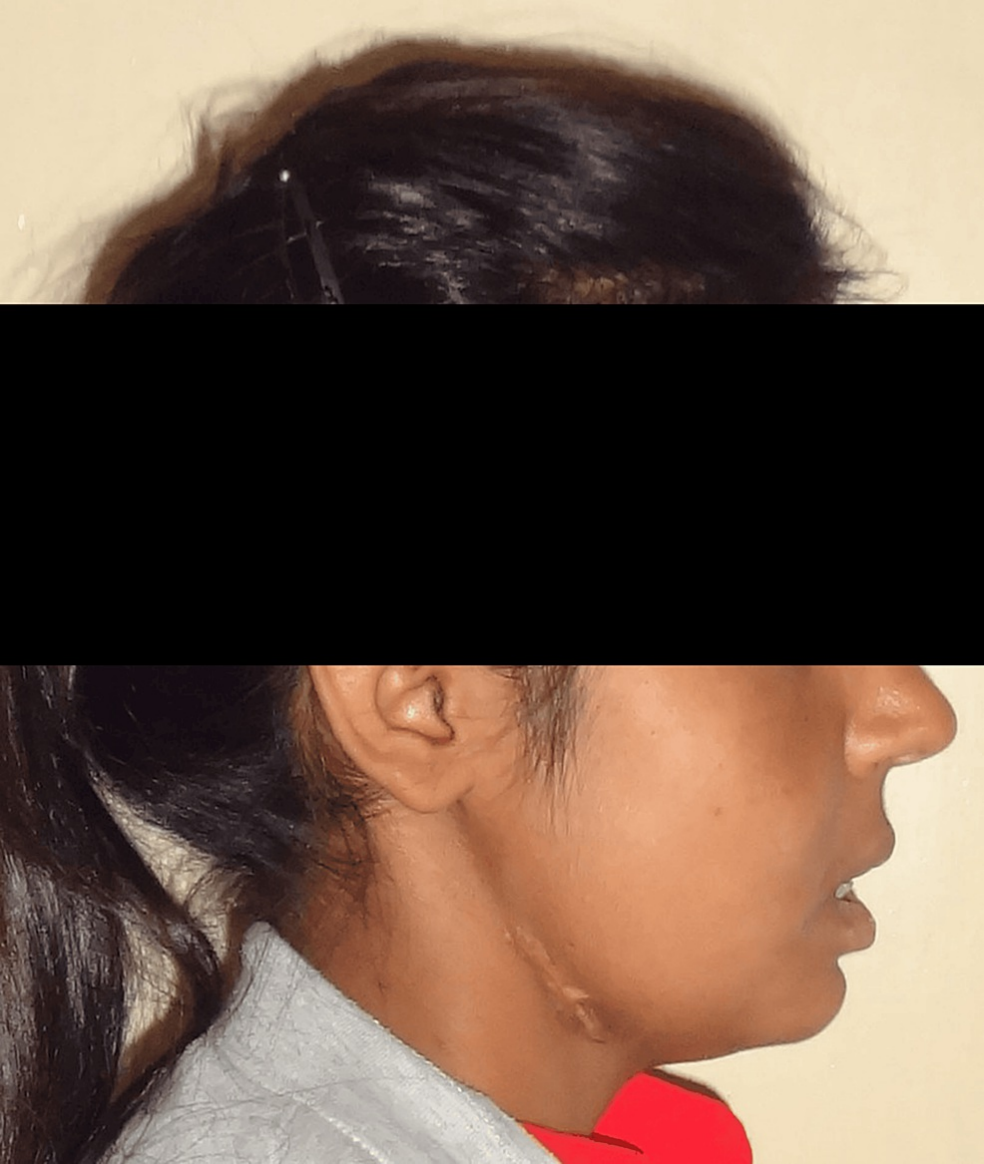
Pretreatment profile photograph.

Considering the relatively young age and concave profile (Figure 3), rapid maxillary expansion (RME) [3] and intrusion of the maxillary right central incisor were proposed simultaneously during the first phase of the treatment to salvage the tooth and expand the maxillary alveolus base utilizing the residual maxillary growth, followed by mandibular first premolar extractions, and correction with a fixed orthodontic appliance during the second phase.

**Figure 4:**
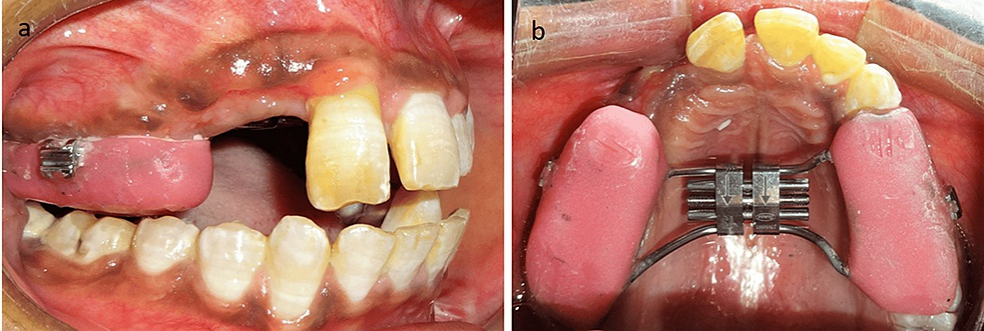
(a) Right lateral view showing the modification of the Hyrax assembly by buccally incorporating molar tubes in the acrylic. (b) Occlusal view of the modified bonded Hyrax assembly.

During fabrication of the bonded Hyrax assembly, a weldable MBT triple molar tube with an auxiliary slot was embedded bilaterally in the acrylic adjacent to the buccal surfaces and parallel to the molar’s buccal cusps of the maxillary first molars (Figures 4A, 4B).

**Figure 5:**
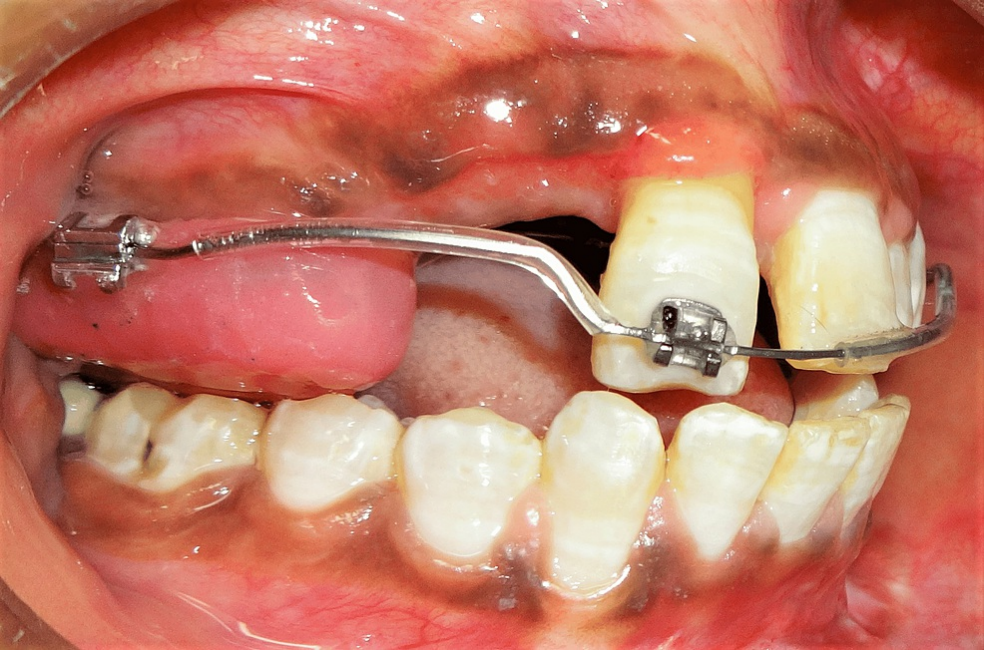
Right lateral view showing the initiation of molar tube-supported intrusion of the incisor.

**Figure 6:**
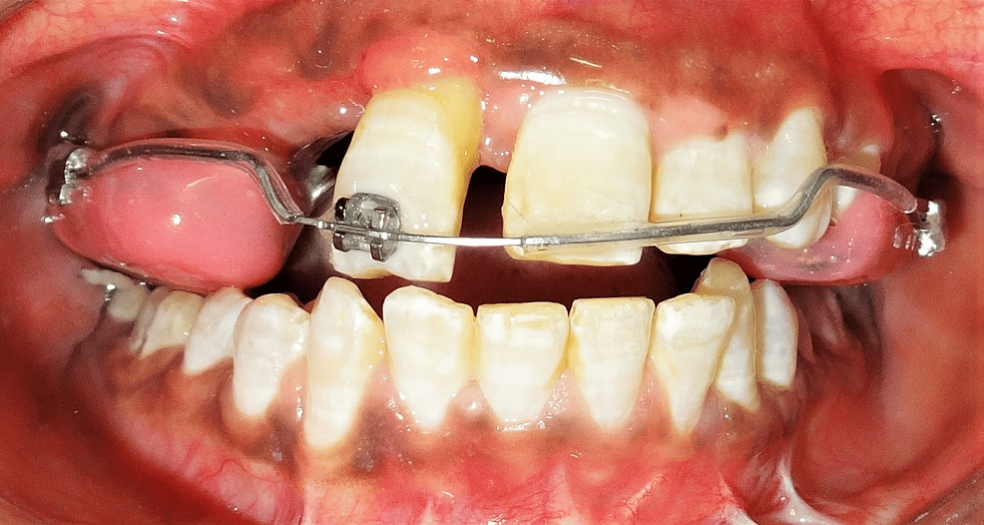
Frontal view showing the initiation of intrusion of the incisor.

After the appliance was cemented, the patient was instructed to activate the appliance twice on the first day, and thereafter once a day for 21 days, creating an opening of 5.75 mm. When sufficient expansion had been achieved with the buccal segments being overcorrected by a half cusp, the screw was locked with a double ligature tie. During this time, orthodontic repositioning of the extrusive maxillary right central incisor was initiated using a light-force 0.017 x 0.025-inch titanium-molybdenum alloy (TMA) intrusion arch (Figures 5, 6).

**Figure 7:**
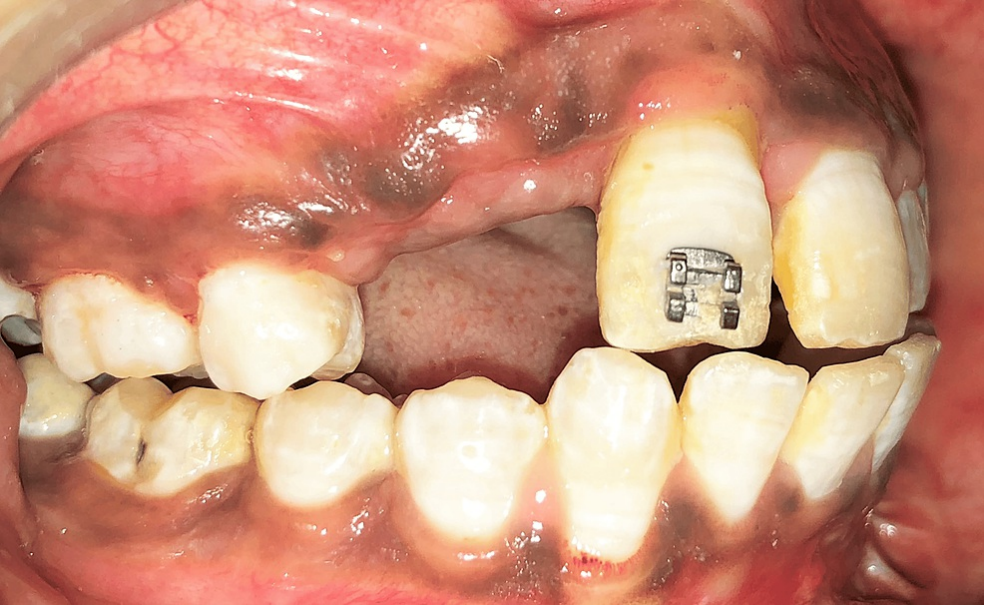
Right lateral view showing accomplished intrusion at the end of the stabilization period.

**Figure 8:**
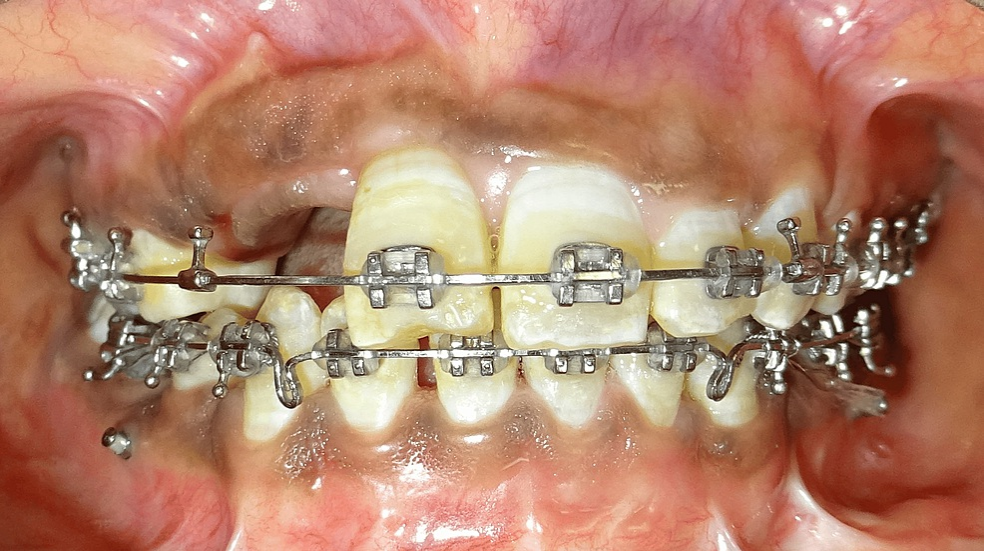
Frontal view showing favorably aligned upper incisor.

At the end of the stabilization period, i.e., after three months, intrusion of the incisor was completed (Figure 7). Considering the severity of the injury, endodontic treatment was contemplated. Stabilization and consolidation of both expansion and repositioned tooth were performed with the existing design of the Hyrax assembly. Further improvement in incisor alignment achieved with a comprehensive fixed mechanotherapy is demonstrated in Figure 8. A fixed prosthesis is planned for the replacement of the missing maxillary right lateral incisor and canine following the completion of the orthodontic treatment.

## Discussion

By virtue of their position and adversely affecting frontal aesthetics, form, and function, upper anterior teeth affected by dentoalveolar traumatic injuries require immediate attention [4]. An individualized treatment approach taking into consideration the biological, functional, esthetic, and economic factors as well as the patient’s expectations is crucial in such cases [5]. The benefits of capitalizing on the orthodontic and orthopedic effects of RME for addressing transverse and anteroposterior discrepancies in young children have been well documented in the literature [6]. The fear of creating a negative profile change due to RME caused by downward and backward rotation of the mandible can be a major concern to many clinicians and patients when planning treatment for a hyperdivergent patient with maxillary constriction. In such patients, using a bonded Hyrax expander with occlusal acrylic coverage prevents further hinge opening of the mandible and increases the vertical dimension by exerting an intrusive force on the maxillary and mandibular teeth [7].

The present patient exhibited maxillary transverse deficiency as the intermolar distance, measured from the cervical margins, was 31.4 mm which was smaller than the average normal width of 35.4 mm [8]. In the present case requiring concomitant expansion and incisor intrusion, using a modified bonded Hyrax assembly with buccally incorporated molar tubes helped achieve significant intrusion of the traumatically extruded incisor during the stabilization phase after RME. Moreover, the posterior bite blocks of the bonded assembly prevented any palatal displacement of the maxillary incisor due to traumatic contact with the mandibular incisors. In this case, TMA wire was chosen for incisor intrusion because of its favorable characteristics, such as high springiness, low stiffness, excellent formability, and efficient working range [9]. This simple modification enables efficient delivery of anterior intrusive force without any reactionary extrusive forces on the molars.

## Conclusions

This simple modification of a Hyrax assembly enables effective intrusion of traumatically extruded upper incisors without compromising the course of RME, thereby reducing the overall treatment time and uplifting the patient’s self-esteem. This also helps capitalize on the advantage of residual growth and simplifies the need for comprehensive fixed orthodontic appliances.

The modified Hyrax expander with embedded molar tubes can be utilized as a versatile interim appliance for simultaneous management of various other orthodontic problems such as crowding, spacing, and incisor proclination without compromising the basic integrity of the bonded assembly, without causing a delay in the overall treatment duration and reducing the complexity of fixed orthodontic therapy in the second phase. This procedure can be adopted during a mixed dentition period as well.
